# Application Progress of Immobilized Enzymes in the Catalytic Synthesis of 1,3-Dioleoyl-2-palmitoyltriglyceride Structured Lipids

**DOI:** 10.3390/foods14030475

**Published:** 2025-02-02

**Authors:** Xing Ni, Ting Feng, Yuyang Zhang, Zhiyuan Lin, Fanzhuo Kong, Xue Zhang, Qiongya Lu, Yani Zhao, Bin Zou

**Affiliations:** School of Food and Biological Engineering, Jiangsu University, Zhenjiang 212013, China; 2212318069@stmail.ujs.edu.cn (X.N.); 13023480269@163.com (T.F.); 2212218043@stmail.ujs.edu.cn (Y.Z.); 2222218077@stmail.ujs.edu.cn (Z.L.); 2222218033@stmail.ujs.edu.cn (F.K.); 2212318042@stmail.ujs.edu.cn (X.Z.); 3046077379@stmail.ujs.edu.cn (Q.L.); 2212418062@stmail.ujs.edu.cn (Y.Z.)

**Keywords:** 1,3-dioleoyl-2-palmitoyltriglyceride, immobilized enzyme, enzymatic synthesis

## Abstract

In recent years, the preparation of OPO (1,3-dioleoyl-2-palmitoyltriglyceride)-structured lipids through immobilized lipase catalysis has emerged as a research hotspot in the fields of food and biomedical sciences. OPO structured lipids, renowned for their unique molecular structure and biological functions, find wide applications in infant formula milk powder, functional foods, and nutritional supplements. Lipase-catalyzed reactions, known for their efficiency, high selectivity, and mild conditions, are ideal for the synthesis of OPO structured lipids. Immobilized lipases not only address the issues of poor stability and difficult recovery of free enzymes but also enhance catalytic efficiency and reaction controllability. This review summarizes the latest advancements in the synthesis of OPO structured lipids using immobilized lipases, focusing on immobilization methods, enhancements in enzyme activity and stability, the optimization of reaction conditions, and improvements in product purity and yield. Furthermore, it delves into the reaction mechanisms of enzymatic synthesis of OPO structured lipids, process optimization strategies, and the challenges and broad prospects faced during industrial applications.

## 1. Introduction

1,3-dioleoyl-2-palmitoyltriglyceride (OPO structured lipids), a major component of fat in human milk, effectively addresses issues such as infant constipation and the absorption of calcium and fatty acids. The National Health Commission of China has approved the use of OPO structured lipids as a nutritional fortifier in infant formula milk powder. As an ideal triglyceride for infant formula, OPO structured lipids have been a research hotspot in this field in recent years. Therefore, the purpose of this review is to understand the reaction mechanisms of enzymatic synthesis of OPO structured lipids, as well as the latest advancements in immobilized lipase-related reaction parameters for the synthesis of OPO structured lipids, providing a reference value for their industrial applications.

### Overview and Synthesis Methods of OPO Structured Lipids

Breast milk is the most ideal food source for infants, containing only 3% to 5% lipids yet providing 50% to 60% of infants’ energy requirements, as well as essential fatty acids [1,2]. Approximately 98% of the fat in breast milk exists in the form of triacylglycerols (TAGs), and their structural distribution determines the physiological functions of breast milk fat. The saturated fatty acids in TAGs are primarily located at the sn-2 position of the glycerol backbone (especially palmitic acid (PA), which accounts for about 70% of the saturated fatty acids at the sn-2 position), while the sn-1 and sn-3 positions of TAGs are mainly occupied by unsaturated fatty acids (such as oleic acid, linoleic acid, linolenic acid, etc.). This structure facilitates the digestion and absorption of breast milk fat by infants [3].

**Figure 1:**
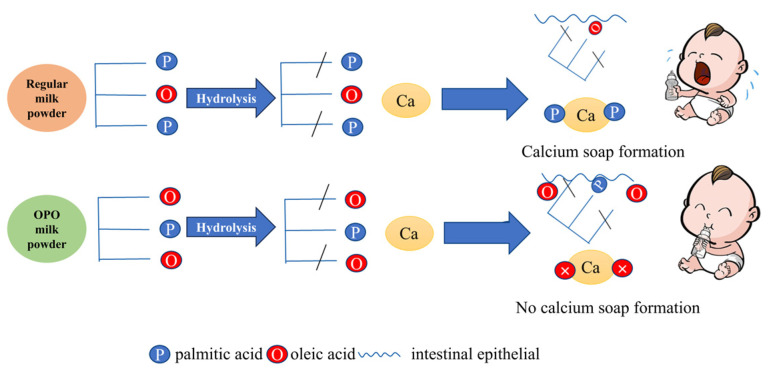
Metabolism map of OPO structured lipids in vivo.

1,3-dioleoyl-2-palmitoyltriglyceride (OPO structured lipids) is the most abundant triglyceride in breast milk, playing a crucial role in the digestion and absorption process of infants and young children, and a high-level sn-2 palmitate diet increased calcium absorption from 42% to 57% [4]. As shown in Figure 1, OPO structured lipids with palmitic acid at the sn-2 position and oleic acid at the sn-1 and sn-3 positions, upon hydrolysis by pancreatic lipase in the gastrointestinal tract, release a large amount of free unsaturated fatty acid oleic acid (OA) and sn-2 monopalmitin (sn-2 MAG). Regular milk powder will form calcium soap, which can cause babies to cry. However, milk powder rich in OPO structured lipids will not form calcium soap, and babies can better absorb the nutrients in the milk powder. The free unsaturated fatty acids do not bind with calcium ions in the intestine to form fatty acid calcium salts, thereby reducing the risk of constipation. Meanwhile, sn-2 monopalmitin serves as an easily absorbed energy source for intestinal cells, promoting rapid growth in infants. Clinical studies on OPO structured lipids have demonstrated its additional functions such as enhancing bone strength, reducing inflammation, and improving memory. Consequently, research on the preparation of OPO structured lipids is receiving increasing attention.

Currently, the main global producers of OPO are Bunge Loders from the United States, Wilmar International from Singapore, Aarhus Karlshamn from Sweden, and so on; China still relies on imports of OPO structured lipids from brands such as Betapol^TM^ from the Netherlands and InFat^TM^ from Israel [5].Therefore, the research and development of OPO structured lipids can promote the upgrading and sustainable development of China’s infant formula industry. At present, the preparation methods of OPO structured lipids are mainly divided into chemical synthesis and enzymatic synthesis. Chemical synthesis utilizes chemical reagents to catalyze the synthesis of OPO structured lipids, but this method involves vigorous reactions and poses risks of chemical solvent residues and environmental pollution [6]; On the other hand, enzymatic synthesis employs lipases as catalysts, offering advantages such as mild reactions, high catalytic specificity, and environmental friendliness, thus exhibiting broader application prospects.

**Figure 2:**
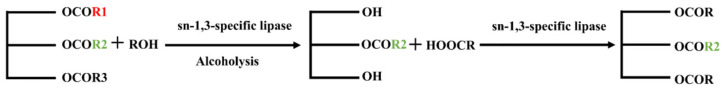
Synthesis of OPO structured lipids via enzymatic alcoholysis.

The enzymatic synthesis of OPO structured lipids mainly includes alcoholysis, transesterification, and acidolysis. In enzymatic alcoholysis, oils rich in palmitic acid (such as tripalmitin (PPP) or lard) are first subjected to alcoholysis with ethanol under the catalysis of specific lipases to generate sn-2 monopalmitin (2-MAG). Subsequently, 2-MAG is esterified with oleic acid to produce OPO structured lipids (Figure 2). Liu et al. [7] used PPP as the raw material to produce 2-MAG through solvent-free enzymatic alcoholysis. They then esterified 2-MAG with ethyl oleate under the catalysis of *Candida* sp. 99-125 lipase, resulting in an OPO yield as high as 85.06%. This method is confronted with challenges in terms of solvent usage, cost, reaction efficiency, the treatment of by-products, and environmental impact. Optimizing the solvent usage, reducing the cost of catalysts and alcohols, enhancing reaction efficiency, minimizing waste generation, and effectively treating these wastes are the key issues that need to be addressed.

**Figure 3:**
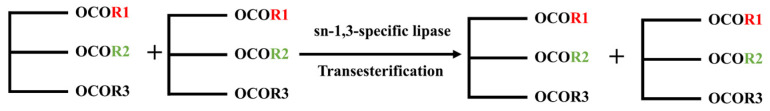
Synthesis of OPO structured lipids via enzymatic transesterification.

Enzymatic transesterification involves the ester–ester interchange reaction between two different triglycerides or between a triglyceride and an acyl ester, catalyzed by sn-1,3-specific lipases, to generate a new triglyceride, namely OPO structured lipids (Figure 3) [4]. Lee et al. [8] used PPP and ethyl oleate as substrates and catalyzed the ester–ester interchange with lipase from *Thermomyces lanuginosus*, obtaining an OPO content of 31.43%. Sarah A et al. [9] utilized soybean oil and PPP as raw materials, catalyzed by the commercial enzyme Novozym 435, ultimately achieving a sn-2 palmitic acid content of 60.84%. Enzymatic transesterification does not generate by-products, but both the substrates and products are triglycerides, which are similar in nature. As a result, product separation is difficult, and the yield is not high. To improve the separation efficiency and purity of the product, strategies such as using excess reactants, removing the product, selecting appropriate solvents and catalysts, and employing methods like distillation, extraction, and crystallization can be applied.

**Figure 4:**
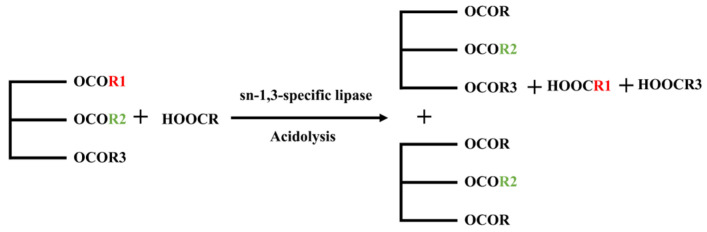
Synthesis of OPO structured lipids via enzymatic acidolysis.

Compared to the multi-step reactions of alcoholysis and the challenging separation of transesterification, acidolysis is more favored by scientists. Enzymatic acidolysis involves the generation of OPO structured lipids through the catalysis of lipases using triglycerides rich in sn-2 palmitic acid and raw materials rich in oleic acid (Figure 4). The substrate triglycerides mainly include tripalmitin, basa catfish oil, lard, palm stearin, etc., while the raw materials rich in oleic acid primarily comprise oleic acid or oleic acid-rich vegetable oils (such as sunflower oil, rapeseed oil, camellia oil, etc.). Zheng et al. [10] used PPP and OA as substrates and performed acidolysis under the catalysis of *Candida lipolytica* lipase, achieving an OPO yield of 46.5%. Zhang et al. [11] catalyzed the reaction between lard and oleic acid at a mass ratio of 1:2 using *Candida lipolytica* lipase and simultaneously added β-cyclodextrin to enhance the yield of enzymatically catalyzed synthesis of OPO structured lipids. After 10 h of catalysis, the final OPO yield was 55.3%. This method facilitates easier product separation, and compared to the separation costs of transesterification and the multi-step reactions of alcoholysis, it is more conducive to large-scale industrial production.

## 2. Research Progress of Lipase Catalysts in the Enzymatic Synthesis of OPO Structured Lipids

### 2.1. Overview of Lipase

Lipases (EC 3.1.1.3), which are widely found in plants, animals (pancreas, liver, and stomach), and microorganisms (bacteria, fungi, and yeast), can catalyze reactions such as acidolysis, esterification, ammonolysis, alcoholysis, and transesterification, making them one of the most extensively used biocatalysts in the field of biotechnology [12,13,14]. Lipases exhibit excellent stability, high enantioselectivity and regioselectivity, as well as broad specificity towards various non-natural substrates, providing vast potential applications for their development [15]. Compared to chemical catalysts, lipases offer significant advantages such as high catalytic efficiency, strong specificity, mild reaction conditions, easy process control, environmental friendliness, and fewer by-products.

### 2.2. Catalytic Mechanism of Lipase in Triglycerides

Although lipases from different sources exhibit certain differences in amino acid sequences, relative molecular weights, and catalytic activities, most lipases are built upon the α/β hydrolase fold, sharing similar three-dimensional structures and catalytic active centers. The active center consists of a catalytic triad composed of serine (Ser), histidine (His), and glutamic acid (Glu) or aspartic acid (Asp) [16,17]. Lipase molecules are composed of hydrophilic and hydrophobic segments, exhibiting a specific phenomenon of interfacial activation. This is mainly because the active center of the lipase is located at the bottom of a V-shaped region that resembles a pocket, and the disulfide bonds above the pocket are connected to amino acid residues to form a “lid” structure that covers the enzyme’s active site [18,19]. The inner surface of the “lid” near the active catalytic site is hydrophobic, while the other side is hydrophilic. The active state of the lipase is achieved through the opening and closing of the “lid”. When the lipase encounters a hydrophobic interface or substrate, the “lid” structure covering the active site opens, exposing the active site of the lipase and allowing the substrate to enter and bind to the active site, thereby triggering the catalytic reaction. This conformational state of the lipase is described as the “active conformation”, and this characteristic is referred to as “interfacial activation”.

The catalytic mechanism of lipases follows the “Ping-Pong Bi-Bi” reaction mechanism. The serine residue in the active site of the catalytic triad is activated through hydrogen bonding with the acidic residue and histidine, attacking the acyl substrate to generate an acyl–enzyme intermediate. This acyl–enzyme intermediate is then attacked by molecules with strong electronegativity and nucleophilic characteristics (such as water or alcohol molecules), ultimately releasing the product and the enzyme. Subsequently, the lipase enters the next round of catalytic reaction [20].

### 2.3. Application of Lipase in OPO Structured Lipids

Sn-1,3-specific lipases are the preferred catalysts for the synthesis of OPO structured lipids and other triacylglycerols (TAGs) and have been a focus of researchers for more than 20 years. These lipases exhibit high selectivity for the sn-1 and sn-3 positions of the glycerol backbone in TAGs. Therefore, when sn-1,3-specific lipases are used as catalysts, fatty acid redistribution occurs only at the sn-1 and sn-3 positions of the triglycerides, while the fatty acid at the intermediate sn-2 position remains unchanged. Studies have shown that sn-1,3-specific lipases exhibit excellent catalytic properties in the synthesis of triglycerides. In recent years, *Rhizomucor miehei* lipase has been the primary lipase used in the synthesis of OPO structured lipids, along with other lipases such as *Rhizopus oryzae* lipase, *Aspergillus oryzae* lipase, *Candida lipolytica* lipase, and *Thermomyces lanuginosus* lipase.

Gu et al. compared the effects of stirred tank reactors and continuous packed bed reactors on the synthesis of OPO through the acidolysis of palm stearin with oleic acid catalyzed by *Aspergillus oryzae* lipase. The results indicated that the continuous packed bed reactor caused less mechanical damage to the lipase and provided a more uniform temperature distribution, showing potential for industrial application [21]. Faustino et al. used *Rhizopus oryzae* lipase to catalyze a solvent-free acidolysis reaction in a stirred batch reactor and compared it with the commercial enzyme Lipozyme RM IM. The results showed that under optimal reaction conditions, about 52% of new TAG (HMFS) was generated after 24 h of reaction, which was comparable to the catalytic effect of the commonly used commercial immobilized lipase (Lipozyme RM IM) [22]. Ghide et al. utilized *Rhizomucor miehei* lipase (RML) as a catalyst to synthesize OPO structured lipids through a two-step enzymatic acidolysis of tripalmitin (PPP) with oleic acid (OA). The research results demonstrated that the sn-1,3 position OA incorporation rate was 55.43% when using *Rhizomucor miehei* lipase for OPO synthesis, compared to 52.87% for the commercial enzyme Lipozyme RM IM, indicating that the catalyst used in this experiment had better catalytic performance [23]. Peng et al. also employed *Rhizomucor miehei* lipase as a catalyst to prepare human milk fat substitutes rich in OPO structured lipids using tripalmitin (PPP) and oleic acid (OA) as raw materials. The research results showed that under optimal reaction conditions, the content of OPO structured lipids in the product was 43.97% [24].

Although sn-1,3-specific lipases are often used as biocatalysts for the synthesis of OPO structured lipids [25], free lipases are prone to inactivation under extreme conditions such as organic solvents, extreme pH values, and high temperatures, limiting their industrial application. These limitations can be overcome by immobilizing the enzymes onto solid supports to enhance their stability and reusability. However, in the acidolysis synthesis process of OPO structured lipids, immobilizing the lipase transforms the catalytic system from homogeneous to heterogeneous. Additionally, the high viscosity and physical properties of the substrates’ tripalmitin and oleic acid lead to substrate diffusion limitations, steric hindrance, and partition effects, which significantly and adversely affect the catalytic efficiency of the reaction. Therefore, selecting appropriate immobilization methods and supports for lipases is a crucial factor influencing the enzymatic synthesis of OPO structured lipids [26,27,28].

## 3. Research Progress of Immobilized Lipase Catalysts in the Enzymatic Synthesis of OPO Structured Lipids

### 3.1. Lipase Immobilization Methods

The immobilization method of lipases is one of the key factors for successful immobilization, directly affecting the enzyme’s activity and the catalyst’s lifespan. Common immobilization techniques for enzymes can be broadly classified into adsorption, entrapment, covalent binding, and crosslinking [29,30,31,32,33].

Adsorption involves binding free enzymes to solid supports through secondary bonds such as hydrogen bonds, electrostatic interactions, van der Waals forces, and hydrophobic interactions, which can be further divided into physical adsorption and ion adsorption [34,35,36,37,38,39]. The advantages of this immobilization method include simple operation, gentle reaction conditions, and generally protecting the enzyme’s active site from damage during the immobilization process, thereby maintaining the stability of the enzyme’s conformation to the greatest extent. However, due to the weak interaction forces between the carrier and the lipase, the enzyme may easily leak from the carrier after multiple uses, resulting in poor operational stability of the immobilized enzyme obtained through adsorption. Neda et al. immobilized lipase on a novel Co^2+^-chelated magnetic nanostructure through physical adsorption, obtaining an immobilized enzyme with high activity, high thermal stability, and reusability. After seven cycles of hydrolyzing 4-nitrophenyl palmitate, the immobilized enzyme still retained 70% of its initial enzyme activity [40]. Aghaei et al. immobilized α-amylase on a calcium silicate carrier (CLA) through physical adsorption. The results showed that the immobilization efficiency of α-amylase reached 93.7%, with an expressed activity of 90.2% of the free enzyme. Moreover, the immobilized enzyme exhibited higher thermal stability and storage stability compared to the free enzyme. After incubation at 80 °C, the activity of the immobilized enzyme CLA was 4.03 times that of the free enzyme. After a 15-day stability test, the residual activity of CLA was 65.3%, while that of the free enzyme was only 49.4%. However, after 10 cycles of reuse, the residual activity of the immobilized enzyme was only 35.9% of its initial activity [41].

The entrapment method refers to a technique where the enzyme is encapsulated within the pores of a polymer carrier. In this immobilization method, there is generally no binding reaction between the enzyme and the carrier, and the process is simple with gentle reaction conditions, thus maintaining the enzyme’s molecular conformation and preserving the activity of the immobilized enzyme. In entrapment immobilization, the enzyme is embedded within a porous polymer structure, allowing small molecules such as substrates and products to freely pass through. Unlike adsorption, entrapment effectively protects the enzyme from direct contact with the reaction medium, thereby minimizing potential inactivation effects due to the properties of the solvent in the medium [42,43,44]. However, this method has limitations due to the small internal pore size of the polymer carrier, which prevents large-molecular substrates from accessing the enzyme’s active site, resulting in restricted internal spatial movement for large-molecular substrates or products, thus limiting its application scope. The pore size limitation directly determines the interaction between the enzyme and the substrate as well as the efficiency of the enzymatic catalytic reaction through multiple aspects such as the entry of the substrate, the spatial activity of the enzyme, the selectivity of the reaction, and the concentration gradient of the substrate in the reaction environment. Choosing an appropriate pore size is crucial for optimizing the enzymatic catalytic performance in the encapsulation method. Additionally, entrapment methods also suffer from disadvantages such as low enzyme loading capacity and enzyme molecule leakage [45]. Karim et al. entrapped carboxymethyl cellulase (CMCase) in agarose gel, and compared to the native enzyme, the immobilized enzyme exhibited superior catalytic properties over a wider range of temperatures and pH values. The operational and storage stability of the immobilized enzyme was also significantly improved, with residual enzyme activities of 50.52 ± 2.5% and 18.94 ± 0.9% for the immobilized and native enzymes, respectively, after 60 days [46]. Xing et al. entrapped Pseudomonas aeruginosa TF-06 in calcium alginate beads and used it for the degradation of patulin in apple juice. The experimental results showed that the immobilized enzyme beads could effectively detoxify patulin in apple juice with a removal rate of 95%, without negatively affecting the quality of the juice [47].

The crosslinking method involves the formation of insoluble aggregated immobilized enzymes through covalent bonding between multifunctional or bifunctional reagents and enzyme protein molecules. Crosslinking preparation methods can generally be classified into three types: (1) the crosslinking agent can react with the enzyme alone; (2) the enzyme is first adsorbed onto the surface of a carrier, followed by crosslinking; (3) new functional groups are introduced onto the carrier surface using multifunctional reagents (such as glutaraldehyde, dextran aldehyde, etc.) and then connected to the enzyme [48]. The immobilized enzymes obtained using this method have a strong bond between the carrier and the enzyme molecules, making them less prone to leakage and exhibiting good reusability. However, this reaction is relatively harsh, resulting in a significant loss of enzyme activity. Moreover, the mechanical strength of the crosslinked enzymes is relatively low, making them prone to breakage. In industrial production, crosslinking enzymes are often used in large-scale reactors and are subjected to physical stresses such as stirring and pumping. Enzymes with low mechanical strength are prone to damage, resulting in decreased reaction efficiency and the need for frequent replacement or repair, thereby increasing costs. Therefore, in immobilization applications, this method is rarely used alone and is often combined with other immobilization methods (such as adsorption and entrapment) to enhance enzyme stability while retaining its activity. Nouri et al. immobilized pectinase onto chitosan-modified magnetic nanoparticle carriers using the green crosslinking agent kefiran polysaccharide. The results showed that after one month of storage, the residual activity of the immobilized enzyme was 1.88 times that of the free enzyme. In addition to improved storage stability, the immobilized enzyme also exhibited higher thermal and pH stability [49]. Xia et al. obtained an enzyme with crosslinked aggregates using glutaraldehyde as a crosslinking agent to produce a novel ionic liquid-modified lipase. The results indicated that the activity of the immobilized enzyme was 5.51 U/mg of protein, and after incubation at 60 °C for 50 min, CRL-FIL-CLEAs still retained 61% of its initial activity. This method provides a new approach for the effective synthesis of immobilized enzymes [50].

The covalent binding method is a technique for obtaining immobilized enzymes through the formation of stable covalent bonds between enzyme protein groups and functional groups on the surface of insoluble carriers. This method enables a stronger bond between the enzyme molecules and the carrier, preventing the enzyme molecules from detaching and thus enhancing the enzyme’s stability under extreme conditions. However, the drawbacks include harsh reaction conditions for covalent binding, complex operational procedures, and alterations to the enzyme’s molecular structure during the immobilization process, resulting in low enzyme activity recovery [51,52,53]. Despite these drawbacks, the covalent bond connection restricts the structural flexibility of the enzyme in the reaction system, preventing protein leaching and denaturation, making the covalent binding method one of the most widely used methods in immobilization applications. Aghaei et al. immobilized lipase from Candida rugosa onto epoxy-activated calcium silicate 30B through covalent binding to obtain immobilized enzyme (LECL). The immobilized lipase exhibited a good hydrolytic activity of 1.85 U/mg during the hydrolysis of olive oil. When applied to the synthesis of biodiesel and isoamyl acetate; LECL also demonstrated excellent catalytic activity, with yields of 91.6% and 95.4% for esters and biodiesel, respectively [54]. Guisan et al. also mentioned in the literature that obtaining multiple functional groups on the activated carrier surface for covalent binding to stabilize the enzyme can result in efficient and robust immobilized biocatalysts [55].

### 3.2. Immobilized Enzyme Carrier for Synthesis of OPO Structured Lipids

In the process of immobilization, the choice of carrier is also crucial for the success of immobilized enzymes, directly affecting the productivity of the enzyme and the lifespan of the catalyst [56,57,58,59,60,61]. By immobilizing enzymes onto suitable solid carriers, the enzyme’s structure is stabilized, making them more resistant to the surrounding environment and largely maintaining their efficacy. Additionally, this facilitates the separation of the catalyst from the products in the reaction medium, becoming a key pathway in the enzyme industry and biomanufacturing applications [62,63,64,65,66,67]. In recent years, many sn-1,3-specific lipases have been studied for the synthesis of OPO structured lipids, among which the commercially immobilized lipases Lipozyme TL IM and Lipozyme RM IM are the most widely used in the enzymatic synthesis of OPO-structured lipids [68]. Wang et al. used the commercial enzymes Lipozyme RM IM (lipase from *Rhizomucor miehei* immobilized on resin) and Lipozyme TL IM (lipase from *Thermomyces lanuginosus* immobilized on silica) as catalysts to prepare structured TAGs rich in OPO and OPL through enzymatic acidolysis. The results showed that the yields of Lipozyme TL IM and Lipozyme RM IM were 35.77% and 46.73%, respectively [69]. Peng et al. mainly used the commercial immobilized enzyme Lipozyme RM IM to study the effect of water molecules on acyl migration and the yield of OMO triacylglycerols through transesterification reactions. The results indicated that a trace amount of water activity improved the yield of OMO by promoting acyl migration [70]. However, the high cost and susceptibility to depletion of these immobilized lipases limit their implementation on an industrial scale. The cost of commercial immobilized lipase is usually high, mainly including the cost of the immobilization process, the cost of enzyme procurement, and stability issues such as the loss of enzyme activity and the degradation of carrier materials.

In recent years, with the increasing research on OPO-structured lipids, commercial immobilized lipases have been unable to meet the needs of researchers, leading to the emergence of more carriers in the study of OPO-structured lipids. Currently, the carriers for immobilized enzymes used in the synthesis of OPO-structured lipids mainly include multi-walled carbon nanotubes, Fe_3_O_4_ nanoparticles, mesoporous molecular sieves, silica microspheres, and resins. For example, Zheng et al. used magnetic multi-walled carbon nanotubes as a carrier to adsorb lipase CLL, and the resulting immobilized enzyme CLL@mMWCNTs exhibited excellent catalytic performance in the enzymatic synthesis of OPO, with activities 1.21 times and 1.40 times that of the commercial lipases RM IM and TL IM, respectively [10]. Ghide et al. immobilized *Rhizomucor miehei* lipase (RML) onto magnetic multi-walled carbon nanotubes (mMWCNTs) to obtain the immobilized enzyme RML-mMWCNTs. Using tripalmitin (PPP) and oleic acid (OA) as substrates, they synthesized OPO-structured lipids through a two-step enzymatic acidolysis, obtaining an effective biocatalyst for the synthesis of OPO-rich structured lipids [71]. Peng et al. crosslinked RML onto magnetic Fe_3_O_4_ nanoparticles using dialdehyde starch. The magnetic nanoparticles maintained the enzyme’s specificity and catalytic efficiency while improving its stability under different reaction conditions. A 43.97% yield of OPO structured lipids was achieved after reacting at 50 °C for 7 h [24]. He et al. immobilized *Thermomyces lanuginosa* lipase (TLL) onto a hydrophobic mesoporous molecular sieve C_18_H_37_-SBA-15 carrier to obtain the immobilized enzyme TLL@C_18_H_37_-SBA-15. Using this as a catalyst for the enzymatic acidolysis synthesis of OPO structured lipids, the results showed a PPP conversion rate of 99.07% and an sn-2 palmitic acid content of 90.09% [72]. Cai et al. covalently bound lipase onto functionalized magnetic silica microspheres for the synthesis of OPO structured lipids. The immobilized enzyme exhibited good stability and reusability, retaining 85% of its initial activity after nine cycles of synthesizing OPO structured lipids [73]. The choice of carrier differs in the synthesis of OPO structured lipids, and a good choice of the immobilized enzyme carrier should provide a suitable and friendly microenvironment for the enzyme [74].

A review of the recent literature on the enzymatic catalytic synthesis of OPO is presented in Table 1. Currently available immobilized enzyme carriers each have their advantages and disadvantages. For instance, while magnetic materials offer relatively convenient recovery, immobilized enzymes on magnetic nanomaterials suffer from poor dispersibility and chemical stability, as well as low enzyme adsorption capacity [75,76,77,78,79,80]. Porous materials, such as molecular sieves [81,82], silica [83,84,85,86,87,88], and metal–organic frameworks [89,90,91,92,93], are ideal carriers for enzyme immobilization due to their large specific surface area, high porosity, and relatively stable mechanical and chemical properties. However, these materials are limited by difficulties in encapsulating large-sized enzymes, the lack of diversity in surface functional groups, weak interactions between the carrier and enzyme, the inability to adjust pore sizes to match those of the enzyme, and the toxicity of metal ions to protein molecules. Therefore, there remains a significant challenge in preparing a suitable immobilized carrier for the enzymatic acidolysis synthesis process of OPO structured lipids [94]. The design of different carrier structures plays a crucial role in enzyme activity, stability, specificity, and other aspects. Thus, finding a more suitable carrier for lipase immobilization remains key to the efficient and green synthesis of OPO structured lipids [72].

## 4. Research Progress of Novel Enzyme Carrier Covalent Organic Framework Materials (COFs)

In the acidolysis process, immobilized lipase (EC 3.1.1.3) catalysts have attracted considerable attention from researchers due to their advantages of mild reaction temperatures, excellent 1,3-regioselectivity, and ease of separation and reuse from the reaction system. However, after lipase immobilization, the catalytic system transitions from homogeneous to heterogeneous, and the internal acyl groups of triacylglycerols in the OPO structured lipid reaction system are prone to self-migration under prolonged heating conditions. Therefore, enhancing the mass transfer performance of high-viscosity substrates in the synthesis process of OPO structured lipids, particularly by eliminating the internal diffusion limitations of reactants within the immobilized enzyme particles to reduce byproducts and accelerate the reaction, is an urgent issue that needs to be addressed.

In light of the numerous side reactions and prolonged reaction times caused by mass transfer limitations in current OPO structured lipid preparation processes, rapid substrate diffusion within immobilized enzyme catalysts is particularly important. Therefore, it is necessary to design the structure of enzyme immobilization carriers to eliminate the impact of intraparticle diffusion limitations on the heterogeneous reaction system of OPO structured lipids. As an emerging ordered crystalline porous polymeric material in the past decade, covalent organic frameworks (COFs) possess unparalleled characteristics compared to traditional materials, including a high specific surface area (~4000 m^2^/g), adjustable pore size (~4.4 cm^3^/g), good food safety, and superior thermal stability. COFs typically have a larger specific surface area than traditional carriers such as molecular sieves due to their ordered pore structure and greater surface area, which provide more active sites for adsorption and reactions, and their thermal stability is generally superior to that of molecular sieves, mainly owing to the stability of covalent bonds and highly tunable structures [107]. In 2017, the Yaghi research group at the University of California summarized the construction methods of COFs with different nanopores in *Science* and highlighted the great potential of COFs with adjustable pore sizes as enzyme protein carriers [108]. In 2018, Chen Yao and colleagues at Nankai University reported in *Angew. Chem. Int. Ed.* a new method for post-grafting lysozyme onto COF mesoporous molecular cages using a covalent approach. The enzyme loading achieved with this method was as high as 22.5 µmol/g (an approximately 20-fold increase), and after 23 h of reaction, the enzyme leakage rate was below 3%, demonstrating the superior cyclic stability of COF-immobilized enzymes [109]. In the same year, Xi Kai and colleagues at Nanjing University reported in *Nature Communications* a series of water-dispersible amino-functionalized nano-COFs for in vivo drug delivery, showcasing the superior safety performance of COFs as carriers [110]. Ma and colleagues at the University of South Florida reported in *JACS* a novel COF material immobilized with *Pseudomonas cepacia* lipase. Compared to traditional metal–organic frameworks (MOFs) and inorganic materials like MCM-41, the superior permeability of the COF pores resulted in a nearly 40% increase in substrate conversion and a reduction in reaction equilibrium time from 900 min to 30 min [111].

In addition, the application of COF materials in the field of enzyme immobilization is becoming increasingly prevalent. For instance, Samui et al. developed a multifunctional reticulated COF for α-amylase immobilization, where the enzyme is anchored on the surface of the flexible COF framework through strong interactions. This not only increases the enzyme loading but also addresses the issue of amylase’s affinity for substrates [112]. Sun et al. designed a dual-pore COF, lipase@COF-ETTA-EDDA, where the lipase is encapsulated in large hexagonal pores and the substrates and products enter and exit through triangular pores, thus enhancing mass transfer rates and achieving higher catalytic efficiency and stability [113]. Cheng et al. aimed to design a carrier that could solve the problem of the lack of specific interactions between the material and the enzyme. They designed a novel covalent organic framework, COF-BTDH, and immobilized pectinase on this new host material through physical adsorption for the extraction of ginsenosides. The immobilized enzyme maintained over 85% of its activity after five cycles of reuse [107]. Wang et al. used glutaraldehyde as a functional reagent to covalently immobilize trypsin onto magnetic COFs (MG@TpPa-1). They successfully achieved a high loading capacity of 268 μg/mg. The prepared MG@TpPa-1–trypsin not only improved the catalytic efficiency of trypsin but also exhibited good reusability and operational stability [114]. Through interactions between COFs and enzymes (such as hydrogen bonds, hydrophilic/hydrophobic effects, π-π interactions, etc.), the structural stability of the enzyme can be protected, and the interfacial activity of the lipase can be stimulated. Zhong et al. fabricated a novel hollow microtubular COF for trypsin immobilization, achieving a high loading capacity of 453 mg/g. Moreover, the TatDha-COF with a hollow microtube structure has a faster mass transfer rate, and its catalytic activity increased by 110% [115]. Due to issues such as low enzyme loading, enzyme leaching, and mass transfer limitations in some immobilization systems, Nada et al. studied a multi-level porous COF with cationic properties and immobilized horseradish peroxidase (HRP) on ACA-COF through physical adsorption. The resulting immobilized enzyme, HRP@ACA-COF, exhibited enhanced enzyme loading and stability [116]. Research on COF-immobilized enzymes has focused on addressing challenges such as mass transfer limitations in enzyme immobilization, weak interactions between the carrier and the enzyme, low enzyme loading, and poor stability, showcasing the potential application prospects of COF materials in the field of enzyme immobilization. Therefore, it is evident that COF materials with diverse structures and adjustable pore sizes, as carriers for enzyme immobilization, will significantly improve the diffusion performance of substrates within enzyme particles.

## 5. The Significance and Prospect of Research on the Synthesis of OPO Structured Lipids Catalyzed by Immobilized Enzymes

Despite the numerous advancements in the research on the catalytic synthesis of OPO structured lipids using immobilized lipases, there are still technical and economic challenges that need to be addressed. Future research efforts should focus on the following aspects: firstly, the development of more efficient and stable carriers for immobilizing lipases to enhance enzyme longevity and reaction continuity. secondly, the exploration of novel enzymatic catalytic reaction systems and the optimization of reaction conditions to further improve the synthesis efficiency and product purity of OPO structured lipids; furthermore, the promotion of large-scale application and industrialization of enzymatic catalytic reactions to reduce production costs and meet market demands; and lastly, the integration of genetic engineering and enzyme engineering technologies to cultivate lipase variants with high catalytic activity, thereby enhancing catalytic performance at the source. Jiang et al. conducted directed evolution on the alginate lyase (FsAly7) from *Flammeovirga* sp. and modified it. The optimal temperature of the mutant mFsAly7 was raised by 5 °C, and its thermal inactivation half-life at 40 °C and 45 °C was increased by 4.4 times and 5.6 times, respectively [117]. Through interdisciplinary research and technological innovation, the synthesis of OPO structured lipids catalyzed by immobilized lipases is expected to achieve broader applications in the future, providing new solutions for the development of food, pharmaceuticals, and other functional products.

## Figures and Tables

**Table 1:** Lipase-mediated production of OPO.

Type	Lipase	Materials	Enzyme Dosage (%)	Temperature (℃)	Time (h)	OPO Yield (%)	Reference
Interesterification	Lipozyme TL IM	PS/ethyl oleate	10	50	3	31.43	[8]
Acidolysis	Lipozyme RM IM	34L-leaf lard/camellia oil FAs	6	45	6	43.72	[95]
Acidolysis	Novozym 435	Tuna oil/ PPP/OA	10	37	1	52.1	[96]
Acidolysis	Lipozyme RM IM	PPP/OA	12	60	4	40.23	[97]
Acidolysis	Lipozyme RM IM	PPP/OA	12	50	4	51.8	[98]
Interesterification	Lipozyme IM-20	PPP/OA	8	40	1	55.2	[99]
Alcoholysis	*Candida* sp. 99-125	PPP/OA	15	45	1.5	65	[100]
Acidolysis	CLL@mMWCNTs	PPP/OA	20	50	2	46.5	[10]
Acidolysis	Novozym 435	PPP/OA	8	60	6	71.22	[101]
Acidolysis	lipase NS 40086	PS/OA/LA	8	60	4	39.2	[69]
Acidolysis	Novozyme 435	Glycerol/PA/OA	4	60	24	19.3	[102]
Interesterification	Lipozyme RM IM	soy oil/palm kernel stearin/PS	10	56	5	23.1	[103]
Acidolysis	Novozym 40086	PPP/FFA	10	40	2	17.96	[104]
Acidolysis	TLL@R-SBA-15	PPP/OA	10	50	8	73.15	[72]
Acidolysis	RML@COF-1	PPP/OA	10	45	5	47.35	[105]
Acidolysis	COF@RML	PPP/OA	10	45	6	51.27	[106]

Abbreviations: Lipozyme TL IM, *Thermomyceslanuginosus* lipase immobilized on silica-gel; Lipozyme RM IM, *Rhizomucor miehei* lipase immobilized on anion-exchange resin; Novozym 435, *Candida antarctica* lipase B immobilized onto a macroporous acrylic polymer resin (Lewatit VP OC 1600); CLL, the lipase from *Candida lypolytica* lipase; Lipozyme IM-20, the lipase from *Rhizomucor miehei* lipase; lipase NS 40086, the lipase from *Rhizomucor miehei*; Novozym 40086, the lipase from *Rhizomucor miehei*; TLL, the lipase from *Thermomyces lanuginosus* lipase; RML, the lipase from *Rhizomucor miehei* lipase; PPP, tripalmitin; PA, palmitic acid; OA, oleic acid; LA, linoleic acid; PS, palm stearin; FFA, free fatty acids.

## Data Availability

No new data were created or analyzed in this study. Data sharing is not applicable to this article.
